# Biogenic and Risk Elements in Wines from the Slovak Market with the Estimation of Consumer Exposure

**DOI:** 10.1007/s12011-017-1157-1

**Published:** 2017-10-07

**Authors:** Magdalena Semla, Pavol Schwarcz, Ján Mezey, Łukasz J. Binkowski, Martyna Błaszczyk, Grzegorz Formicki, Agnieszka Greń, Robert Stawarz, Peter Massanyi

**Affiliations:** 1 Institute of Biology, Pedagogical University of Cracow, Podbrzezie 3, 31-054 Krakow, Poland; 2 Department of EU Policies, Faculty of European Studies and Regional Development, Slovak University of Agriculture, Trieda Hlinku 2, 949 01 Nitra, Slovak Republic; 3 Department of Fruit Growing, Viticulture and Enology, Faculty of Horticulture and Landscape Engineering, Tulipánová 7, 949 76 Nitra, Slovak Republic; 4 Department of Animal Physiology, Slovak University of Agriculture, Trieda Hlinku 2, 949 01 Nitra, Slovak Republic

**Keywords:** Biogenic metals, DRV, MOE, PTWI, Slovakia, Wine

## Abstract

Wine consumption delivers macroelements and microelements necessary for the proper metabolism. On the other hand, wine can be an important source of toxic metals. The aim of this study was to estimate the concentrations of Ca, Cd, Cu, Fe, Hg, Mg, Ni, Pb, and Zn in the Slovak and non-Slovak wines. The concentration of metals was evaluated with respect to the type, the alcohol content, and the age of Slovak wine. The general scheme of concentrations found was as follows Ca > Mg > Fe > Zn > Pb > Cd > Ni > Cu > Hg. The type of wine and the alcohol content do not have a significant impact on metal concentrations. Also, the age of wine has no influence on the mean concentration of metals, except for Zn. Metal concentrations in Slovak and non-Slovak wines indicate similar contents of metals, except for Ni. The contribution to both dietary reference values (DRVs) and provisional tolerable weekly intake (PTWI) evaluations in the Slovak wine suggested low dietary exposure to Ca, Cu, Fe, Mg, Ni, Zn, Cd, Hg, and Pb, respectively. However, we do not suggest that the consumption of all Slovak wines is healthy. The maximum Pb concentrations in Slovak wines exceed the maximum permitted level proposed by the European Commission. This might be proved by the results of the margin of the exposure (MOE) value evaluation in the samples containing the maximum Pb concentrations, showing a high risk of CKD and SBP in high and extreme consumption groups.

## Introduction

Slovakia, thanks to its relatively warm climate, is a very dynamic and efficient wine producer and exporter. The total surface of Slovak vineyards is about 20,000 ha across the six main wine regions, mainly located in the southern part of the country [1]. Growing wine industry contributes to the increase of wine consumption among the local population up to 18.3% of all the alcohol consumption which places the Slovak Republic on the 41st place of wine consumption in the world [2]. Wide Slovak wine consumption entails the need for careful control of wine quality and its impact on human health. Moreover, metals in wine may have a negative effect on the organoleptic properties of wine [3].

The popularity of wine is connected to its numerous bioactive molecules and natural elements which play an important role in health and prevention of diseases. Wine also constitutes the source of vitamins, macronutrients, and micronutrients, including metals [4]. Metals perform various functions in an organism, e.g., they participate in the synthesis and maintenance of the nucleic acid structure; they are parts of the basic compounds like proteins, lipids, hemoglobin, and enzymes essential for the functioning of the organism, and they play a fundamental role in the neuromuscular excitability and water-electrolyte or acid-base balance [5–9]. Furthermore, metals contained in wine may be potentially toxic to humans. Exposure to heavy metals may lead to serious poisoning, circulatory system diseases, nervous system disease, and cancer [10]. Some metals in low concentrations are fundamental to the human organism, while at higher doses, they may pose a health risk [7, 8].

Metal concentrations in wine may have an impact on the quality of the wine. The bioavailability of metals may be an important factor in governing the fermentation performance by wine yeasts. Furthermore, metal concentrations are often responsible for imparting the salty character in wines [11]. Metal ions play an important role in oxidation and reduction reactions, which, in turn, affect the browning of wine, cloudiness, turbidity, and astringency [4].

Still, poorly understood is the mineral content of wine which affects its toxicity and human health. Daily consumption of wine in moderate quantities may be a source of mineral elements, required by the human organism [12]. On the other hand, high wine consumption may contribute to a large fraction of heavy metal intake. Additionally, the evaluation of the content of metals in the consumed wines is important due to their impact on the sensory attributes [11]. Therefore, the monitoring of metals in wine is advisable.

The aim of this study was to assess the concentration of calcium (Ca), cadmium (Cd), copper (Cu), iron (Fe), mercury (Hg), magnesium (Mg), nickel (Ni), lead (Pb), and zinc (Zn) in the Slovak wine. We examined the concentration of metals depending on the type of wine (red, rose, and white), the alcohol content (≥ 12 and < 12%), and the age of wine (≤ 2 and > 2 years). The correlations between metal concentrations were also checked. In addition, the concentrations of metals in the Slovak wines were compared with the metal content in the non-Slovak wines. Finally, for Cd, Pb, and Hg metals, the contribution to provisional tolerable weekly intake (PTWI) in the Slovak wine was also estimated. Furthermore, we assess the margin of the exposure (MOE) value for normal, high, and extreme Slovak wine consumption.

## Material and Methods

Metal concentrations were determined in 56 Slovak wines available on the Slovak market and 21 from other countries (Australia, Bulgaria, the Czech Republic, Hungary, Italy, Moldova, Spain, Switzerland, the USA). The Slovak wines came from four regions and were divided into three groups according to the type of wine (white, red, rose), the alcohol content (≥ 12 and < 12%), and the age of wine (≤ 2 and > 2 years).

All the metal analyses were determined in the laboratory of the Institute of Biology in the Pedagogical University of Cracow in Poland. All the samples (1 mL each) were mineralized in hot nitric acid (65%, Baker Analyzed, JT Baker, Deventer, the Netherlands) with the open mineralizer (Velp Scientifica DK-20, Usmate, Italy). The mineralized samples were used to prepare 10 mL solutions with ultrapure water (18.2 MΩ cm, Millipore Integral 3, Merck Millipore, Darmstadt, Germany). Concentrations of Cd, Ca, Cu, Fe, Pb, Mg, Ni, and Zn were measured with flame atomic absorption spectrometer (PerkinElmer AAnalyst 200, PerkinElmer, Waltham, MA, USA).

Hg concentrations in wine samples (150 μL each) were determined with cold vapor atomic absorption spectrometer (MA-2, Nippon Instrument Corporation, Tokyo, Japan). Each sample was analyzed twice, and the mean value was calculated. The limits of the analytical methods were calculated for each metal according to the protocol described by Fleming et al. [13]. The validity of the whole procedure was checked against the certified reference material (ERM-CE195 CRM-bovine-blood, Institute for Reference Materials and Measurements, Belgium; BCR-463-tuna fish, Joint Research Centre, Institute for Reference Materials and Measurement, Belgium; SRM1577b-bovine liver, National Institute of Standards & Technology, USA, Table 1). For Mg and Ni, the accuracy of the method was checked by ran repeated samples and spikes every ten samples studied. All the recoveries were found within the range of 90–110%.

**Table 1:** Parameters of the analytical procedure: recoveries for certified reference materials (CRMs, *n* = 10) with relative standard deviation (RSD) of 10 replicates

Metals	Wavelength *λ* (nm)	LoD (mg/L)	LoQ (mg/L)	Recovery (%)	RSD (%)
Ca	422.7	0.0642	0.514	108.2	6.6
Cd	228.8	0.0621	0.010	91.6	4.3
Cu	324.8	0.0876	0.035	99.6	2.7
Fe	248.3	0.005	0.415	101.6	6.2
Hg^a^	253.7	–	0.27	97.1	2.6
Mg	285.2	0.00015	0.017	–	–
Ni	232.00	0.006	0.013	–	–
Pb	217.0	0.1039	0.107	93.7	6.2
Zn	213.9	0.0170	0.024	106.2	5.3

For Cd and Pb, ERMCE195 CRM were used; for Hg, BCR-463 CRM was used and for other elements, SRM1577b; no appropriate CRM for Mg and Ni were found

*LoD* limits of detection (in solution), *LoQ* limits of quantification established for wine samples

^a^LoQ value for Hg expressed as a nanograms per liter

For Cd, Pb, and Hg metals, the contribution to PTWI in the Slovak wine was estimated per consumer (70 kg of body weight) according to the formula:

$$ \mathrm{PTWI}\  \mathrm{contribution}\ \mu g/ kg\  bw/ week=\frac{M_{Me}\times 0.15}{m}\times 7\ \mathrm{days} $$

*M*_*Me*_: mean concentration of metals in milligrams per liter
0.15: 150 mL wine/one glass/one person
*m*: 70 kg/one person

The estimated daily intake (EDI) provides an estimate of expected dietary exposure. EDI was calculated for the Slovak wine consumption for Ca, Cu, Fe, Mg, Ni, and Zn metals [14]. The daily intake of metals depends on the metal concentration in wines (*M*_*me*_) and the daily wine consumption (150 mL/per person/per day). In addition, the body weight (70 kg/per person) of the human can influence the tolerance of contaminants.

$$ \mathrm{EDI}\ \mathrm{mg}/\mathrm{kg}\ \mathrm{bw}/\mathrm{day}=\frac{MMe\times 0.15}{m} $$

Panels on Dietetic Products, Nutrition, and Allergies developed a scientific opinion on dietary reference values (DRVs) for the European population. To estimate the DRVs, this opinion includes the average requirement (AR) and adequate intake (AI). For Ca, Fe, and Zn, the DRVs in the Slovak wine were calculated on the basis of AR [15–17]. For Cu and Mg, the DRVs were calculated on the base of AI [18, 19].

### Statistical Analysis

Since the data met the demands of the parametric tests, we carried out the one-way ANOVA or *t* test for particular categorical factors: type of wine, alcohol content, and age of the wine. We used Pearson correlation factors to evaluate the potential relationships between metals’ concentrations and between concentration and age. The significance level was set at 0.050. All the calculations and analyses were done with Excel 2016 for Mac (Microsoft) and Statistica 12 (StatSoft).

## Results

### Metal Concentrations in Slovak and Non-Slovak Wines

The general scheme of ascending concentrations of metals in the studied Slovak wines was as follows Ca > Mg > Fe > Zn > Pb > Cd > Ni > Cu > Hg. In the non-Slovak wines, the general scheme of ascending concentrations was as follows: Ca > Mg > Fe > Zn > Ni > Cd > Pb > Cu > Hg. Mean concentrations of Ca, Mg, Fe, Zn, Cd, Pb, Cu, and Hg from the Slovak wine are comparable to the non-Slovak wine. Only the Ni concentration was significantly higher in the non-Slovak wines (*p* < 0.01). Furthermore, in three samples of the Slovak wine and one sample of the non-Slovak wine (from Hungary), the Pb concentrations were over 200 μg/L (Table 2).

**Table 2:** Concentrations of metals (mg/L) in Slovak (*n* = 56) and non-Slovak (*n* = 21) wine with statistical comparison (*t* test) between groups studied

Metals	Slovak wine				Non-Slovak wine				*t*	*p* value
	Mean	SD	Minimum	Maximum	Mean	SD	Minimum	Maximum		
Ca	2.64	1.46	0.00	9.71	2.17	1.07	0.44	3.79	1.36	0.18
Cd	0.07	0.04	0.00	0.15	0.07	0.04	0.00	0.13	− 0.26	0.79
Cu	0.03	0.02	0.00	0.08	0.03	0.02	0.00	0.08	− 0.48	0.63
Fe	0.35	0.17	0.10	0.99	0.39	0.20	0.10	0.79	− 0.89	0.37
Hg	0.001	0.001	0.00	0.01	0.00	0.00	0.00	0.00	0.79	0.43
Mg	0.66	0.84	0.06	5.97	0.65	0.37	0.09	1.28	0.04	0.97
Ni	0.04	0.05	0.00	0.23	0.09	0.11	0.00	0.51	− 2.73	0.01
Pb	0.08	0.06	0.00	0.25	0.06	0.06	0.00	0.21	1.19	0.24
Zn	0.19	0.20	0.01	1.44	0.21	0.16	0.02	0.62	− 0.38	0.70

### Influence of the Type of Wine, Alcohol Content, and Age of Wine on Metal Concentrations

The type of wine did not have a significant impact on metal concentrations in the analyzed wine (Table 3). The trend showed that among all the metals tested, white wine was characterized by the highest concentrations of Mg, Pb, and Zn. The highest concentrations of Ca and Fe were observed in the rose wine. Moreover, the highest concentrations of Cd and Ni metals were observed in the red wine. The Cu concentrations were similar in the white and red types of wine. Moreover, our results indicate Hg elements in the Slovak wine. The average Hg concentration was 0.001 mg/L in all the tested types of wine (Table 4).

**Table 3:** Statistical differences in mean metal concentrations (*n* = 56) in respect of the type of wine (ANOVA), alcohol content, and age of wine (*t* test)

Metals	Type of wine (white, red, rose)		Alcohol content (%) (≥ 12; < 12)		Age of wine (years) (≤ 2; > 2)	
	*F*	*p*	*t*	*p*	*t*	*p*
Ca	0.44	0.65	− 1.22	0.23	0.80	0.43
Cd	2.05	0.14	0.87	0.39	0.64	0.53
Cu	1.62	0.21	− 0.76	0.45	− 1.08	0.28
Fe	1.36	0.27	0.10	0.92	− 0.81	0.42
Hg	0.93	0.40	− 0.26	0.80	0.49	0.63
Mg	0.54	0.59	− 1.48	0.14	1.22	0.23
Ni	0.71	0.50	− 0.69	0.50	1.66	0.10
Pb	0.62	0.54	− 0.75	0.46	− 0.21	0.84
Zn	0.13	0.87	1.04	0.30	− 2.17	0.03

**Table 4:** Metal concentration in Slovak wine (mean ± SD, mg/L, *n* = 56) depending on the type of wine, alcohol content, and age of wine

Metal	Type of wine			Alcohol content (%)		Age of wine (years)	
	White (*n* = 37)	Red (*n* = 7)	Rose (*n* = 12)	≥ 12 (*n* = 40)	< 12 (*n* = 16)	≤ 2 (*n* = 42)	> 2 (*n* = 14)
Ca	2.72 ± 1.58	2.29 ± 1.32	2.82 ± 0.99	2.49 ± 1.12	3.02 ± 2.07	2.73 ± 1.58	2.37 ± 1.00
Cd	0.06 ± 0.04	0.09 ± 0.03	0.07 ± 0.04	0.07 ± 0.04	0.06 ± 0.04	0.07 ± 0.04	0.06 ± 0.05
Cu	0.03 ± 0.02	0.03 ± 0.01	0.02 ± 0.02	0.03 ± 0.02	0.03 ± 0.03	0.03 ± 0.02	0.03 ± 0.02
Fe	0.32 ± 0.15	0.40 ± 0.14	0.41 ± 0.29	0.35 ± 0.17	0.35 ± 0.16	0.34 ± 0.17	0.38 ± 0.15
Hg	0.001 ± 0.001	0.001 ± 0.001	0.001 ± 0.001	0.001 ± 0.001	0.001 ± 0.001	0.001 ± 0.001	0.001 ± 0.001
Mg	0.74 ± 0.98	0.50 ± 0.36	0.49 ± 0.58	0.56 ± 0.45	0.92 ± 1.40	0.74 ± 0.93	0.42 ± 0.41
Ni	0.04 ± 0.05	0.06 ± 0.04	0.03 ± 0.05	0.04 ± 0.04	0.05 ± 0.06	0.05 ± 0.05	0.02 ± 0.03
Pb	0.09 ± 0.06	0.06 ± 0.08	0.08 ± 0.05	0.08 ± 0.06	0.09 ± 0.06	0.08 ± 0.07	0.08 ± 0.06
Zn	0.20 ± 0.24	0.17 ± 0.09	0.18 ± 0.07	0.21 ± 0.22	0.15 ± 0.11	0.16 ± 0.09	0.29 ± 0.35

Alcohol content in the Slovak wine did not have a significant impact on the metal concentration in the analyzed samples (Table 3). The general trend in the Slovak wine was that the wines containing more than 12% of alcohol characterized with higher Ca, Mg, Ni, and Pb concentrations compared to other wines. The content of Fe, Cu, and Hg was the same in both analyzed contents of alcohol (Table 4).

Furthermore, metal concentrations in the analyzed wine do not depend on the age of the wine, except for Zn. The wines more than 2 years of production have significantly higher concentrations of Zn than the 2-year or less-year-old wines (Table 3).

### Correlations of Metal Concentration

Positive correlations between Cu-Fe (*r*_*s*_ 0.29, *p* < 0.05) and Cu-Zn (*r*_*s*_ 0.43, *p* < 0.05) concentrations in the Slovak wine were found. Moreover, the Pb concentration was positively correlated with the Zn content (0.28). Also, a positive relationship between age of the wine and Zn concentration (*r*_*s*_ 0.38, *p* < 0.05) was noted. The type of wine and alcohol content in the analyzed wine did not show correlations between the tested metals.

## Discussion

In this study, all the tested metals were present in the Slovak wines. The type of wine and alcohol content did not have an impact on the metal content in the Slovak wine, contrary to the age of wine which has an impact on the Zn concentration. Furthermore, the Slovak wine is characterized by similar metal content as the non-Slovak wine, except for Ni. Lower Ni concentration in the Slovak wine was observed.

### Metal Concentrations

Generally, the results showed that the Slovak wines contain macronutrients (Ca, Mg) and micronutrients (Cu, Fe, Ni, Zn) which play an important role in health and prevention of diseases [4, 7, 8]. The metal content may be influenced by many factors. Grape varieties, climate, soil, wine-making practices, pH, and yeasts are some of the factors, which may impact the metal content in wines. The detected high concentrations of Mg and Ca metals can be caused by supplementing with growth media which stimulates the yeast growth [20]. Fertilizers and pesticides (mainly fungicides) applied during the growing season of vines may affect, in the tested wine, the presence of elements, like Cd, Cu, Fe, Pb, and Ni. Moreover, the location of the vineyards in close proximity to road traffic and industrial areas or the wine production and storage may have an impact on the Slovak wine production [12]. Furthermore, the substance contained copper sulfate, used to spray the wines to prevent mildew, which may be the source of Cu in wine [21]. The presence of Pb metal in the Slovak wine may be caused by the existence of this metal in some glass bottles, which may lead to wine contamination. It can be observed especially in decorative, crystal bottles in which the concentration of lead is very high. The longer the wine is stored in these bottles, the higher the Pb concentration detected [22]. Moreover, the presence of Hg metal in the Slovak wine may be indicated with air, water, and soil pollution or industrial area [23]. Furthermore, we found that the type of wine and the alcohol content do not affect metal concentration. Also, the age of wine had no influence on the metal level, except for Zn. Higher concentrations of Zn in the Slovak wines more than 2 years of production can be affected by the production process and wine aging [21]. Only a few moderately strong correlations between metal concentrations in the Slovak wine were found. Positive relationships between Cu-Fe and Cu-Zn elements may be associated with the production methods. Cu, Fe, and Zn metals are very important for the efficient alcoholic fermentation and alcohol production in yeast biomass. During fermentation, the concentrations of these metals are favorable for yeasts as they are required for prosthetic metallo-enzyme activation [24]. Moreover, a strong relationship was detected in Zn and Pb concentration which may be associated with the use of pesticides and fertilizers containing Pb and Zn compounds. The application of those chemical substances during the growing season of vines leads to the increase in the amounts of these metals in wine [12]. The relationship between the age of wine and Zn concentrations, found in this study, can be associated with long-term storage of wine in galvanized containers rich in Zn compounds [21].

We noted that metal concentrations in the Slovak and non-Slovak wines indicate similar contents of metals, except for the Ni element. High Ni concentration in the non-Slovak wine may suggest that Ni contamination can be derived from stainless steel storage tanks and from the pigments of bottles during the storage of wines [25]. On the other hand, when compared to the data gathered in literature, the Ca, Fe, Mg, and Zn concentrations, generally, reach lower values in the Slovak wine as compared to the European ones (Table 5). This observation can be attributed to soil parameters which can significantly affect the composition of grapes and wine [27, 28]. In addition, we noted that Cd concentration in the Slovak wines is higher than in the Czech, Cretan, and Spanish wines (Table 5). The Cd pollution may vary greatly with the geographic, demographic, and the social-economic profile of a region [29]. The highest Cd pollution in Slovakia may be associated with the metallurgical industry and agriculture [30]. In addition, due to the Slovakia’s close vicinity of Poland, the main sources of cadmium industrial emissions in this area are in the Upper Silesian Industrial Region of Poland [31]. Generally, the concentrations of Cu, Ni, and Pb in the Slovak wines fall within the range noted in the European wines. According to the regulation issued by the European Commission [32], the maximum permitted level for the Pb content in wines is 200 μg/L for wines produced from the 2001 to 2015 fruit harvest. The maximum level of Pb measured in the Slovak wines is 250 μg/L which is higher than the permitted level (Table 2). These wines are sold in the Slovak market which may constitute a health threat. Metal toxicity depends on the absorbed dose, the route of exposure, and the duration of exposure. Exposure to high levels of Pb can cause brain damage, paralysis, anemia, and gastrointestinal symptoms. Furthermore, the mechanism of Pb toxicity causes significant changes in various biological processes such as cell adhesion, intracellular and intercellular signaling, protein folding, apoptosis, enzyme regulation maturation, ionic transportation, and release of neurotransmitters [33].

**Table 5:** Concentration of metals (mg/L) in wine from four countries in Europe

Metals	Czech wine^a^	Cretan wine^b^	German wine^c^	Spanish wine^d^
Ca	40–100	–	58–200	12–241
Cd	0.000055–0.0033	0–0.006	–	0.00–0.019
Cu	0.012–6.827	0.2–0.6	0.02–0.71	0.00–3.1
Fe	0.9–5.2	4.7–12	0.4–4.2	0.4–17.4
Hg	–	–	–	–
Mg	7.8–138	–	56–105	50–236
Ni	0.019–0.034	0–2.3	–	0.005–0.079
Pb	0.010–1.253	0.018–0.42	–	0.001–0.096
Zn	–	0.3–31	0.3–1.5	0.00–4.63

^a^[26]

^b^[21]

^c^[24]

^d^[24]

Moreover, the Hg concentration in our study cannot be compared with European wines because literature does not show Hg in European wine. However, the Hg concentration found in the studied wine may be indicated with soil and air pollution [28].

### Consumer Risk Assessment

EDI and DRVs were calculated for Ca, Cu, Fe, Mg, Ni, and Zn, because the metals occur in the body at high concentrations. The comparison of EDI with respect to tolerable daily intake (TDI) for Ca, Cu, Fe, Mg, and Zn indicated that daily intake of one glass of the Slovak wine, containing mean and maximum concentrations of these metals, provides less than 0.01% of TDI and does not pose a threat to human health (Table 6). The maximum daily intake of Ni corresponds to 35% of the TDI (Table 6). The compounds of Ni are considered as carcinogens [36]. The estimation of the TDI of Ni has been recently reduced from 8 to 2.8 μg/day [26]. The DRVs are the complete set of nutrient recommendations and reference values, such as population reference intakes, the average requirement, adequate intake level, and the lower threshold intake. The EFSA Panel on Dietetic Products, Nutrition, and Allergies [15–19] estimated DRVs for Ca, Cu, Fe, Mg, and Zn. The contribution to DRV evaluation in the Slovak wine suggested a low dietary exposure for Ca, Cu, Fe, Mg, and Zn (Table 6). Also, the contribution to DRVs for elements like Zn and Cu indicates that consumed in excess, the Slovak wine does not pose a threat to human health.

**Table 6:** Estimated daily intake (EDI) and contribution to the dietary reference values (DRVs) of metals in Slovak wine

Metals concentration (mg/L)	EDI/RDI (mg/kg bw/day)^a^	TDI (mg/kg bw/day)^b^	Contribution to DRVs (%)^c^
Ca			
Mean 2.64	0.006	10,000	0.001
Max. 9.71	0.02		0.003
Cu			
Mean 0.03	0.0001	10	0.01
Max. 0.08	0.0002		0.013
Fe			
Mean 0.35	0.001	18	0.0003
Max. 0.99	0.002		0.03
Mg			
Mean 0.66	0.001	350	0.0003
Max. 5.97	0.013		0.004
Ni			
Mean 0.4	0.0001	0.0028	–
Max. 0.23	0.001		
Zn			
Mean 0.19	0.0004	40	0.003
Max. 1.44	0.0031		0.024

^a^EDI values calculated on the basis of 150 mL consumption (one glass) per person (70 kg) per day

^b^Tolerable daily intake (TDI) for Cu, Ni, and Zn [34]; recommended daily intake (RDI) for Ca, Fe, and Mg [35]

^c^For Ca, Fe, and Zn calculated based on average requirement (AR) [15–17]; for Cu and Mg calculated based on adequate intake (AI) [18, 19]

Due to the Cd, Hg, and Pb trace amounts in organisms, the PTWIs for these metals were calculated. The PTWI is estimated by international scientific committee dealing with contaminants and additives in food. Cd, Hg, and Pb PTWIs are reported by the European Food Safety Agency [37]. The PTWIs of other metals studied are not currently officially regulated by law or norms [38]. We assessed the Cd, Hg, and Pb contribution to PTWI (Table 7) in the Slovak wine (150 mL/one person/day). The estimated contribution to PTWI did not exceed the values proposed by the European Commission. Moreover, the contribution to PTWI evaluation in the Slovak wine suggested relatively low dietary exposure to heavy metals from the Slovak wine.

**Table 7:** The mean concentration of Pb, Cd, and Hg in Slovak wine and contribution to the provisional tolerable weekly intake value (PTWI μg/kg bw/week) calculated for 70 kg person

Metals	mg Cd or Hg or Pb per 150 mL wine	μg Cd or Hg or Pb per one person per week	Contribution to PTWI (%)	PTWI (μg/kg bw/week)
Cd	0.01	1.05	15	7^b^
Hg	0.0002	0.015	0.375	4^b^
Pb	0.01	1.2	4.8	25^a^

^a^Taken from [37]

^b^Taken from [38]

Based on the European Food Safety Agency report [37, 38] on the harmfulness of Cd in food, risk assessment based on the MOE value is recommended. MOE is the ratio between a defined point on the dose-response curve for the adverse effect and the estimated intake of food [39]. Systolic blood pressure (SBP) and chronic kidney disease (CKD) are treated as the most sensitive endpoints used in MOE approach. The European opinion concludes that MOE value of 10 or more brings no significant risk of clinical effects on SBP and change in the CKD. The risk of MOE greater than 1.0 is very low [37]. We assess the MOE for normal consumption (10 L/per capita/per year), high consumption (30 L/per capita/per year), and extreme consumption (50 L/per capita/per year) of the Slovak wine [40]. Based on the MOE evaluation (calculated on the basis of mean metal concentrations), the consumption of Slovak wine poses no high risk of CKD and SBP (Table 8). Additionally, we noted that in the samples containing maximum concentrations of Pb, the risk of CKD and SBP is high, especially for high and extreme consumption groups. Given the magnitude of the MOEs, the possibility of an effect on the kidneys and cardiovascular system in consumers with extreme daily consumption of Slovak wine cannot be excluded. For the maintenance of health, great deals of preventative measures should be taken to avoid consumption with food of potentially toxic metal ions.

**Table 8:** Estimated MOEs for different endpoints by the intensity of Slovak wine consumption

	Pb concentration (μg/L)	MOE—normal consumption	MOE—high consumption	MOE—extreme consumption
Nephrotoxicity				
	Wine (Pb mean 81.0)	0.5–1.6	0.5–1.3	0.4–1.2
	Wine (Pb max. 250.0)	0.5–1.3	0.4–0.9	0.3–0.7
Cardiovascular effects				
	Wine (Pb mean 81.0)	1.2–3.8	1.1–3.2	1.1–2.8
	Wine (Pb max. 250.0)	1.1–3.2	0.9–2.1	0.8–1.6

Margin of exposure (MOE) values calculated for normal consumption (10 L/per capita/per year), high consumption (30 L/per capita/per year), and extreme consumption (50 L/per capita/per year) [40]

## Conclusions

It can be concluded that moderate consumption of Slovak wines may contribute to the daily dietary intake of essential elements and Slovak wines. The occurrence of Cd, Ca, Cu, Fe, Pb, Mg, Hg, Ni, and Zn in various types of wine produced in the Slovakia region was confirmed. The metal contents in the Slovak wines were most often similar to the concentration of metals in the non-Slovak wines. The type of wine and the alcohol content have no effect on the levels of metals in the Slovak wine. The age of wine has an impact only on the Zn concentration. The Slovak wines of more than 2 years of production have significantly higher concentrations of Zn than 2-year or less-year-old ones, which can be affected by the production process and wine aging. We concluded that the estimated daily intake of one glass of Slovak wine containing mean and maximum concentrations of Ca, Cu, Fe, Mg, and Zn provides less than 0.01% of TDI. Only EDI for Ni may be dangerous to human health. The contribution to DRV evaluation in the Slovak wine suggested a low dietary exposure for Ca, Cu, Fe, Mg, and Zn. The PTWI evaluation in the Slovak wine suggested relatively low dietary exposure to heavy metals in the wine. MOE evaluation indicates that average consumption of Slovak wine poses no high cardiovascular and nephrotoxicity threat. However, the maximum concentration of Pb measured in the Slovak wines exceeds the maximum permitted level proposed by the European Commission. For those cases, the higher risk of CKD and SBP for high and extreme consumption groups is possible.
